# The prognostic value and immune landscaps of m6A/m5C-related lncRNAs signature in the low grade glioma

**DOI:** 10.1186/s12859-023-05386-x

**Published:** 2023-07-04

**Authors:** Ran Li, Haiyan Chen, Chaoxi Li, Yiwei Qi, Kai Zhao, Junwen Wang, Chao You, Haohao Huang

**Affiliations:** 1 Department of Neurosurgery, Tongji Hospital, Tongji Medical College, Huazhong University of Science and Technology, Wuhan, 430030 China; 2 Department of Ophthalmology, General Hospital of Central Theatre Command of People’s Liberation Arm, Wuhan, 430070 China; 3 Department of Neurosurgery, General Hospital of Central Theatre Command of People’s Liberation Arm, Wuhan, 430070 China; 4 General Hospital Of Central Theater Command and Hubei Key Laboratory of Central Nervous System Tumor and Intervention, Wuhan, China

**Keywords:** RNA methylation, Long non-coding RNA, Prognostic signature, Immune landscape, Low grade glioma

## Abstract

**Background:**

N6-methyladenosine (m6A) and 5-methylcytosine (m5C) are the main RNA methylation modifications involved in the oncogenesis of cancer. However, it remains obscure whether m6A/m5C-related long non-coding RNAs (lncRNAs) affect the development and progression of low grade gliomas (LGG).

**Methods:**

We summarized 926 LGG tumor samples with RNA-seq data and clinical information from The Cancer Genome Atlas and Chinese Glioma Genome Atlas. 105 normal brain samples with RNA-seq data from the Genotype Tissue Expression project were collected for control. We obtained a molecular classification cluster from the expression pattern of sreened lncRNAs. The least absolute shrinkage and selection operator Cox regression was employed to construct a m6A/m5C-related lncRNAs prognostic signature of LGG. In vitro experiments were employed to validate the biological functions of lncRNAs in our risk model.

**Results:**

The expression pattern of 14 sreened highly correlated lncRNAs could cluster samples into two groups, in which various clinicopathological features and the tumor immune microenvironment were significantly distinct. The survival time of cluster 1 was significantly reduced compared with cluster 2. This prognostic signature is based on 8 m6A/m5C-related lncRNAs (GDNF-AS1, HOXA-AS3, LINC00346, LINC00664, LINC00665, MIR155HG, NEAT1, RHPN1-AS1). Patients in the high-risk group harbored shorter survival times. Immunity microenvironment analysis showed B cells, CD4 + T cells, macrophages, and myeloid-derived DC cells were significantly increased in the high-risk group. Patients in high-risk group had the worse overall survival time regardless of followed TMZ therapy or radiotherapy. All observed results from the TCGA-LGG cohort could be validated in CGGA cohort. Afterwards, LINC00664 was found to promote cell viability, invasion and migration ability of glioma cells in vitro.

**Conclusion:**

Our study elucidated a prognostic prediction model of LGG by 8 m6A/m5C methylated lncRNAs and a critical lncRNA regulation function involved in LGG progression. High-risk patients have shorter survival times and a pro-tumor immune microenvironment.

**Supplementary Information:**

The online version contains supplementary material available at 10.1186/s12859-023-05386-x.

## Introduction

Glioma is the most lethal primary intracranial neoplasm, with a reported overall survival of approximately 14–16 months [1]. The incidence rate of glioma is surmised at 3–4 per 100,000 population worldwide and continues to rise as human life expectancy prolongs [2]. In the case of a new diagnosis, the standard treatment consists of surgery followed by concurrent radiotherapy and temozolomide therapy with additional adjuvant temozolomide treatment. More than half of low grade gliomas(LGG) could progressively evolve into high grade gliomas. Despite recent advances in the treatment of glioma, overall survival remains poor and thus requires new treatment strategies [3]. The update of the WHO classification suggests that the role of molecular phenotyping is of enormous importance for the diagnosis and treatment of glioma [4]. Therefore, the development of molecular phenotyping prognostic risk models provides guidance for screening and discrimination of high-risk patients and might contribute to improving clinical outcomes.

RNA modifications have emerged as pivotal post-transcriptional regulators of gene expression programs, boasting over 170 different types of modifications [5]. N6-methyladenosine (m6A) is the most prevalent chemical epigenetic modification in mRNA post-transcriptional modifications, which is a dynamic and reversible methylation modification at the adenosine N6 position, comprising recognition, methylation, and demethylation [6]. Dynamic m6A modifications are involved in the formation and maintenance of cancer stem cells thereby manipulating cancer progression and treatment resistance [7]. The m6A demethylase ALKBH5 promotes the proliferation and invasion of glioblastoma stem cells by maintaining the expression level of FOXM1 [8]. 5-Methylcytosine (m5C) is a wide-ranging mRNA modification that acts on the untranslated regions (UTRs) of mRNA transcripts to exert regulatory effects including RNA export, ribosome assembly, and translation [9]. Loss of m5C methyltransferase NSUN5 in gliomas correlated with long-term survival of glioma patients [10]. Epigenetic RNA methylation modifications are inextricably linked to tumor proliferation and invasion.

Long non-coding RNAs (lncRNAs) are multifunctional RNAs with structures similar to mRNAs, including poly-A tails and promoter structures, that regulate gene expression through either the editing of RNA, splicing of pre-mRNA, association with chromatin modifiers, or the abrogation of miRNA-induced repression [11]. A variety of lncRNAs have been studied for their engagement in glioma initiation and progression, including H19, Malat1, CRNDE, XIST, HOTAIR, and SOX2OT [12]. m6A is not only the most prevalent modification in mRNAs but also found in long non-coding RNAs [13]. The m6A reader YTHDF2 plays a crucial role in maintaining the stemness of glioma stem cells by stabilizing RNA expression in glioma stem cells [14]. LncRNA NKILA is regulated by the m5c methyltransferase NSUN2 to increase m5C levels, which promotes NKILA interaction with YBX1 for regulating the progression of cholangiocarcinoma [15]. Therefore, m6A and m5C methylated lncRNAs are robustly correlated with tumor development.

However, the exploration of the biological functions of m6A/m5C-related lncRNAs in low-grade gliomas remains scarce due to the limitations of our understanding of lncRNAs. In this article, we will focus on the biological significance of m6A and m5C modifications of lncRNAs in low-grade gliomas. By analyzing 926 glioma samples, we found that the expression of m6A/m5C-related lncRNAs signatures correlated significantly with glioma survival and immunity.

## Methods

### Data collection and processing

Access the TCGA (The Cancer Genome Atlas) and CGGA (Chinese Glioma Genome Atlas) data platforms to download and arrange the transcriptome profiles of LGG (https://portal.gdc.cancer.gov and http://www.cgga.org.cn/). The corresponding clinical information of glioma patients was also downloaded to match the RNA-seq data. Collate the data by ‘Annoprobe’ R package and filter the lncRNA expression data profile from it. The RNA-Seq data of 105 brain normal control tissues from the Genotype Tissue Expression (GTEx) Project were downloaded and collated from the UCSC XENA database (TCGA TARGET GTEx combined cohort, https://xenabrowser.net/). LGG somatic mutation data in TCGA were analyzed and visualized using the R package ‘maftools’[16].

### mRNAs–lncRNAs co-expression network and differential expression level of m6A/m5C-related lncRNAs

Based on the previous report studies [5, 17], a list of 39 m6A/m5C-related genes was yielded (Additional file 2: Table S1). Then, the finding of m6A/m5C genes correlated lncRNAs in TCGA and CGGA cohorts separately by spearman correlation analysis with a setting like absolute value of correlation coefficient > 0.5 and p value < 0.001. The univariate cox regression analysis was used to obtain m6A/m5C-related lncRNA harboring prognostic value from TCGA and CGGA datasets. Subsequently, the intersection of m6A/m5C methylation-related lncRNAs in TCGA and CGGA cohorts to obtain highly correlated lncRNAs. Then we generate gene co-expression network maps using Cytoscape software (Version: v3.9.0) to analyze the results of m6A/m5C spearman correlation analysis [18]. The results of differential analysis of m6A/m5C-related lncRNA gene expression between the expression data of GTEx normal brain samples and LGG expression data in TCGA were presented in a heatmap.

### Consensus clustering glioma cases by m6A/m5C-related lncRNAs

The expression data of correlated lncRNAs were analyzed using the R package “ConsensusClusterPlus”, in which optimal clusterNum was set to 2, resulting in 2 clusters [19]. Subsequently, the distribution of the 2 clusters was visually displayed using the Principal Component Analysis (PCA) method.

### Survival analysis

Survival analysis for the 2 categorical variables was performed by K–M curve analysis for comparison, with statistical differences defined as < 0.05. In another way, patients' survival differences were analyzed by survival status in a 2-category distribution.

### Immune landscapes assessment

ESTIMATE algorithm, Timer algorithm, and Cibersort algorithm were used to evaluate immune scores and immune cell infiltration in 2 classified sample species [20, 21]. Frequently detected 29 immune checkpoint expression data were extracted from LGG gene expression profiles for differential expression comparison across clusters or risk groups [22].

### Functional enrichment analysis

In order to explore differentially expressed genes (DEGs) of different clusters or risk groups, the R package “limma” based on the TCGA dataset was used with the criteria of | log2(FC)|> 1, p < 0.05, and FDR < 0.05 [23]. Gene enrichment analysis (GSEA) was performed to fathom the differentially functional enrichment pathways of samples in distinct clusters. DEGs in the different risk group were opted as candidates for Gene Ontology (GO) analysis and Kyoto Encyclopedia of Genes and Genomes (KEGG) analysis based on the R package ‘clusterProfiler’ and ‘enrichplot’ for visualization [24, 25].

### Tumor-associated somatic mutations and copy number variations analysis

Tumor-associated somatic mutations analysis of 2-classified samples was performed utilizing the ‘maftools’ package. Copy number variation (CNV) evaluations were conducted across clusters or risk groups [26].

### Prognostic signature constructed by m6A/m5C-related lncRNAs

The matched clinical data were first collated. And then the 14 relevant lncRNAs screened were set as independent variables using least absolute shrinkage and selection operator (LASSO) regression analysis, where the method was set as Cox survival analysis to reduce the variables to fabricate a prognostic model. The respective risk score in each tumor sample was calculated by combining correlation coefficients and gene expression level of obtained m6A/m5C-related lncRNAs with such formula (Risk score = ${\sum }_{i=1}^{n}\mathrm{Coefi}*\mathrm{Expi}$, where Coefi means the correlation coefficients, Expi is the FPKM value of each m6A/m5C-related lncRNAs).

### The predictive value and accuracy assessment of the risk model

Receiver operating characteristic (ROC) curve analysis, calibration curve, and time-ROC analysis was used to assess the predictive power and precision of the models [27]. Univariate and multivariate Cox regression analyses were used to assess the value of risk models for clinical application.

### 2.10 In vitro validation assays

#### Cell transfection and siRNA plasmids

The human glioma cell lines U87 and U251 were purchased from ATCC (Manassas, VA). Cells were maintained in DMEM (Gibco, USA) supplemented with 10% fetal bovine serum (Gibco, USA) and 1% penicillin plus streptomycin (Gibco, USA) and incubated in a humidified incubator (37 °C, 5% CO_2_).

The sequences of siRNA were designed based on the NCBI Reference Sequence: NR_037194.1 using the siRNA Wizard software 3.1 (InvivoGen). Sequences of siRNAs were as follows. LINC00664 siRNA #1 GGTGATGACAGAATTGTAACA. siRNA #2 GTGACTGTTCTATTCATCATA. siRNA #3 GTTCAAATGGGACATATCTTA. Si-NC GAGGCGAATCGAATTAGATAT. LINC00664 siRNAs were transfected by using Lipofectamine 2000 (Invitrogen, USA) according to the manufacturer’s instructions.

#### Quantitative real-time PCR

Total RNA from U87 and U251 cells was extracted using a Trizol kit (Servicebio, China) and assessed for purity and concentration. The RNA was reverse transcribed into complementary DNA (cDNA). Quantitative RT-PCR was performed on a PCR machine (ABI Q1, CA, USA) with SYBR Green Master Mix (Yeasen, shanghai, China). Primers were adopted as follows: LINC00664, forward, 5′-TGCCTGTTCTCAGGGAAGAT-3′, reverse, 5′-CAGGCAGAGGACTCACATCA-3′. GAPDH, forward, 5′-AATGGGCAGCCGTTAGGAAA-3′, reverse, 5′-GCGCCCAATACGACCAAATC-3′.

#### CCK8 assay to determine cell proliferation

Firstly, glioma cells were evenly inoculated in 96-well cell plates. After glioma cells were attached to the wall, small interfering RNA to silence LINC00664 was transfected except for the control group. Subsequently, the cells were incubated with CCK8 reagent for 2 h at 22 h, 46 h, and 70 h after the transfection. Finally, the cell proliferation was determined by measuring the absorbance at 450 nm under the microplate reader (BD Biosciences, USA).

#### Trans-well assay to verify the migration ability of glioma cells

The culture apparatus used in the transfer well experiments was a transfer well migration champer (8um size; Corning, USA). After Si-LINC00664 for 24 h, an equal number of each group of cells 5*10^4 were seeded in the upper chamber and DMEM medium with 30% serum concentration was placed in the lower chamber as an attractant. After 24 h of incubation, the upper chamber was fixed, stained, and photographed under a microscope (OLYMPUS BX53).

#### Wound healing assay to assess migration capacity

Glioma cells transfected with GFP were uniformly grown in a 6-well plate and left to form a dense cell layer at the bottom. Then, vertical scribing with a 200 ul microinjection tip to form a scratch. After taking pictures under the microscope, the cells were cultured with DMEM serum-free medium for 24 h and then photographed again. Finally, the distance from the edge of the scratch to the growing edge of the cells was measured to assess the migration capacity of the cells under different conditions.

#### Statistical analysis

All statistical analysis was performed on R Studio (version 4.1.1) and GraphPad Prism (version 9.0). Student’s t-test was performed for two-group comparisons. For comparisons among more than two groups, the Wilcoxon test and one-way ANOVA were utilised respectively for non-parametric and parametric data. p ≤ 0.05 was considered statistically significant.

## Results

### Analysis of m6A/m5C highly correlated lncRNAs

**Fig. 1:**
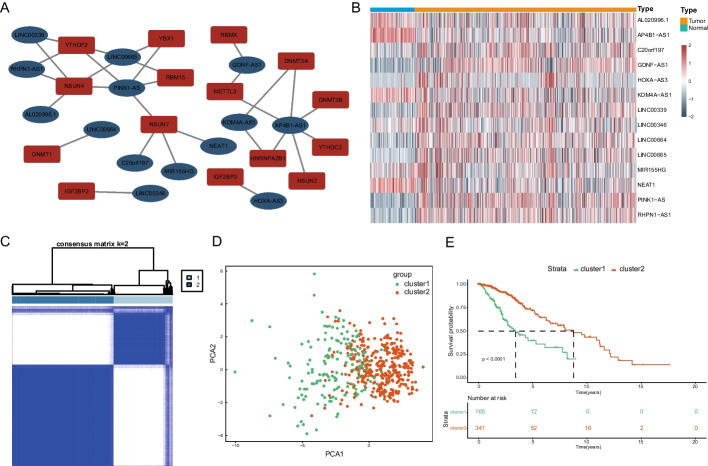
Screening and clustering of m6A/m5C-related lncRNAs in Low Grade Gliomas. **A** The co-expression network of m6A/m5C regulators and related lncRNAs. **B** The heatmap of differential expression of 14 screened lncRNAs with prognostic value between 105 brain normal control tissues from the GTEx dataset and 509 LGG tumor tissues from the TCGA database. **C** Consensus matrix of the consensus clustering based on the 14 screened m6A/m5C-related lncRNAs for optimal k = 2 in 509 LGG tumor tissues from the TCGA cohort. **D** Principal component analysis (PCA) of cluster1 and cluster2. **E** Kaplan–Meier curve of overall survival time between cluster1 and cluster2 (p < 0.0001)

The flow chart of this study was present in Additional file 1: Figure S1. By Spearman analysis, adjusting the correlation coefficient to 0.5, we found 190 correlated lncRNAs with potential prognostic value in the TCGA cohort and 95 correlated lncRNAs in the CGGA cohort (Additional file 1: Figure S2A). The TCGA and CGGA m6A/m5C-related lncRNA data sets were intersected to obtain 14 highly correlated lncRNAs that are representative of the majority of cases (Additional file 1: Additional file 1: Figure S2A, Additional file 2: Table S2). Then, we incorporated the intersecting lncRNAs and related m6A/m5C genes to produce co-expression network maps by Cytoscape software (Fig. 1A). By joining the normal sample data in the GTEx database, we found that these 14 highly correlated lncRNAs were differentially expressed in healthy individuals and LGG cases (Fig. 1B).

### Identification of two clusters of LGG cases by consensus clustering and survival analysis

The LGG cases in the TCGA cohort were classified into two clusters by clustering analysis with the best K = 2 consensus matrix (Fig. 1C). The PCA computation method was performed to display the characteristics of the two clusters from the TCGA cohort (Fig. 1D) or CGGA cohort (Additional file 1: Figure S2B), which were found to be visually distinguishable between these samples. Subsequently, we performed survival analysis on the two clusters of cases and found that the survival time of cluster 1 was significantly reduced compared with cluster 2, both in the TCGA (Fig. 1E) and in the CGGA cohort (Additional file 1: Figure S2C).

### Analysis of oncological differences between the two clusters of LGG cases

**Fig. 2:**
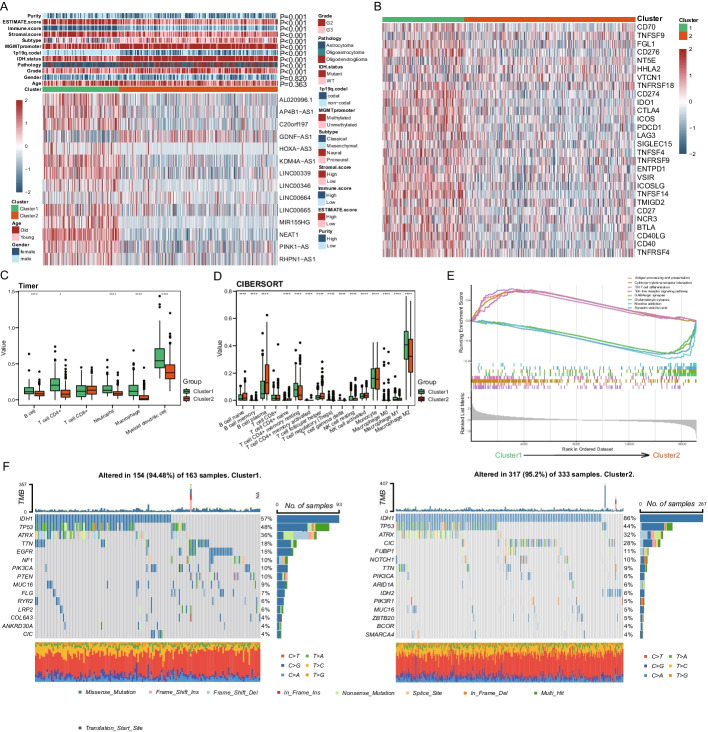
Clinical characteristics, tumor immune landscapes and tumor mutational burden among clustering subgroup in the TCGA-LGG dataset. **A** Heatmap of the clinicopathological features between the two distinct clusters for the TCGA-LGG, the relative expression levels of respective lncRNAs ware plotted in heat map after normalization by using “scale” function. **B** Heatmap of the differential expression status of 29 immune checkpoint genes between cluster1 and cluster2. **C**, **D** Box plots showing differences in the infiltration of immune cells estimated using TIMER2 and CIBERSORT respectively. The Kruskal–Wallis test was used to determine the statistical significance of the difference between cluster1 and cluster2. **E** GSEA analysis displayed key pathways in cluster1 and cluster2. **F** Waterfall plots showing the distribution of the top 15 most frequent somatic mutation in the two clusters, the upper bar graph shows TMB

Through heat map visualization, we showed the differences in expression levels of 14 highly relevant lncRNAs and the clinicopathological features between the two clustered samples (Fig. 2A). Interestingly, we found that cases in cluster 1 had fewer IDH mutations, 1p19q co-deletions, and MGMT promoter methylation both in the TCGA (Fig. 2A, Additional file 2: Table S3) and in the CGGA cohort (Additional file 1: Figure S2D, Additional file 2: Table S5). In addition, it was found that the ESTIMATE score, immune score, and stromal score of cluster 1 were higher compared with cluster 2 (Fig. 2A). Then, the clusters were then analyzed for differences in immune checkpoint expression levels, where we found that immune checkpoints, including CD276, CD40, IDO1, CTLA4, LAG3, PDCD1, TNFSF14 and et al., were significantly upregulated in cluster 1 (Fig. 2B). Furthermore, the two clusters were remarkably different in immune infiltrating B cells, CD4 + T cells, and macrophages by immune cell infiltration analysis with Cibersort and Timer methods (Fig. 2C, D and Additional file 1: Figure S2E, F). By GSEA enrichment analysis, it was found that cluster 1 was highly enriched in immune related pathway, like Antigen processing and presentation, Cytokine–cytokine receptor interaction, Th17 cell differentiation, Toll—like receptor signaling pathway, while cluster 2 was enriched in Glutamatergic synapse, GABAergic synapse, Synaptic vesicle cycle, Nicotine addiction (Fig. 2E). Mutation landscape analysis showed that the highly mutated genes both in clusters 1 and 2 were mainly IDH1, TP53, and ATRX, while cluster 1 had a higher mutated EGFR and PTEN gene compared with cluster 2 in the TCGA cohort (Fig. 2F).

### Constructing and evaluating the risk model by m6A/m5C-related lncRNAs

**Fig. 3:**
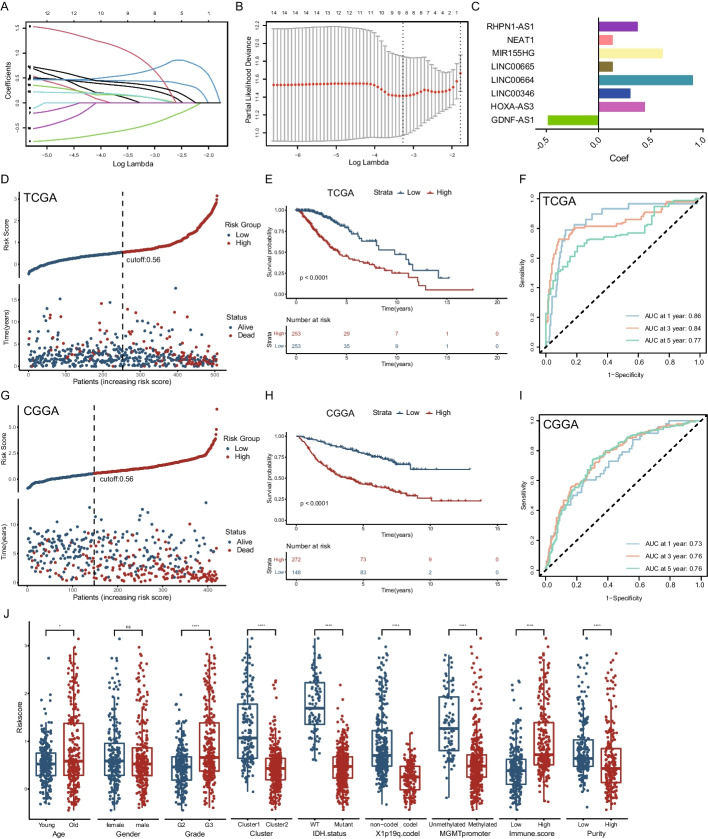
Construction of m6A/m5C-related lncRNAs prognostic signature. **A**, **B** 8 m6A/m5C-related lncRNAs were constructed by using LASSO cox regression. **C** Bar plot shows the correlation coefficient of screened 8 m6A/m5C-related lncRNAs. (D, G) The distributions of risk scores, overall survival status and the expression of 8 m6A/m5C-related lncRNAs in the TCGA-LGG and CGGA-LGG. **E**, **H** Kaplan–Meier curve of OS time of high-risk group and low-risk group in the TCGA-LGG and CGGA-LGG cohort. **F**, **I** ROC curve of the risk score at 1-, 3- and 5-years’ follow-up in the TCGA-LGG and CGGA-LGG cohort. **J** Differences in the low- and high-risk group between various clinicalpathological features. The Wilcoxon test was used to determine the statistical significance

Firstly, LASSO regression analysis was performed to reduce the variables for risk model construction with 14 lncRNAs narrowed down to 8 lncRNAs (Fig. 3A–C). These 8 lncRNAs were combined to build our risk model: GDNF-AS1, HOXA-AS3, LINC00346, LINC00664, LINC00665, MIR155HG, NEAT1, RHPN1-AS1. Subsequently, we grouped patients according to risk scores and found a significantly lower probability of survival among patients in the high-risk group from the TCGA cohort (Fig. 3D, Additional file 2: Table S4) and CGGA cohort (Fig. 3G, Additional file 2: Table S5). Next, a K–M curve was applied to analyze the survival differences between the different risk groups, verifying that patients in the high-risk group had shorter survival times (Fig. 3E, H). Furthermore, we used time-ROC curve analysis to confirm that our model had promising predictive power in survival prediction for both the TCGA (AUC at 1/3/5 years respectively: 0.86, 0.84 and 0.77, Fig. 3F) and CGGA cohorts (AUC at 1/3/5 years respectively: 0.73, 0.76 and 0.76, Fig. 3I). The risk scores were in agreement with previous clustering analyses and provided good discriminatory functions in terms of tumor molecular characteristics, such as fewer IDH1 mutations, 1p19q co-deletion, and MGMT methylation in the high-risk group (Fig. 3J, Additional file 1: Figure S4A).

### The value of risk models compared with other clinical factors in applications

**Fig. 4:**
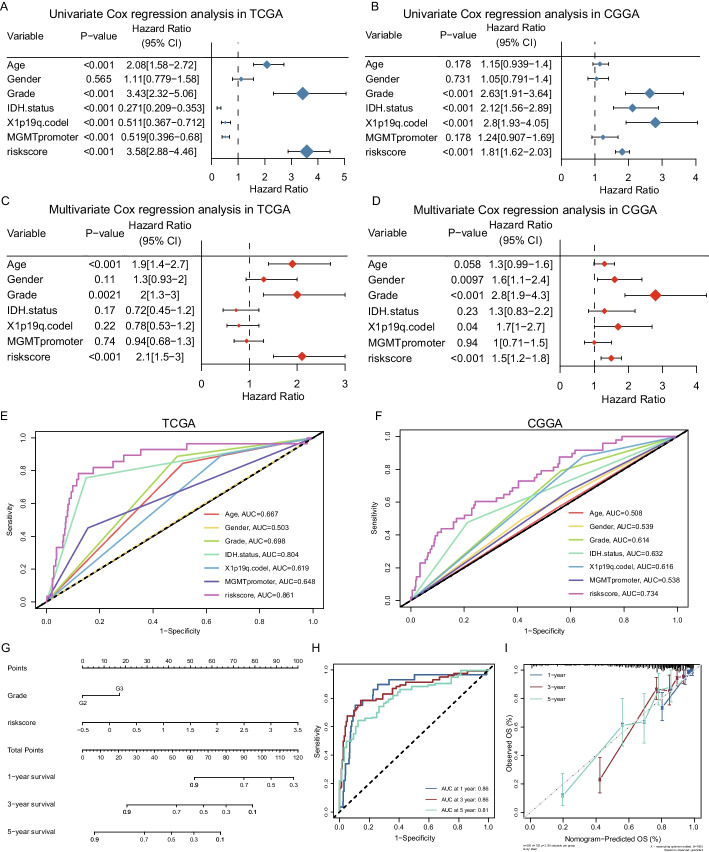
Cox regression analysis and comprehensive nomogram for m6A/m5C-related lncRNAs prognostic signature. **A–D** Forest plots showing univariate and multivariate Cox regression analysis for OS in the TCGA-LGG patients and CGGA-LGG patients. **E**, **F** The ROC curve of the risk score and other clinical characteristics in the TCGA-LGG and CGGA-LGG cohort. **G** The comprehensive nomogram predicting the clinical outcomes of LGG patients with 1-, 3- and 5-year survival based on the TCGA-LGG cohort. **H**, **I** ROC curve and calibration plots for predicting the 1-, 3- and 5-year OS based on the TCGA-LGG cohort

Univariate Cox regression analysis revealed that our risk score was as favorable a prognosis judgment as other commonly used clinical prognostic indicators such as grade, IDH status, 1p19q co-deletion, and MGMT promoter methylation (Fig. 4A, B). Then, through multivariate COX regression analysis, we verified that the risk scores took a better prognostic predictive power compared with molecular characteristics in different cohorts (Fig. 4C, D). Using ROC curves to analyze the degree of predictive accuracy, we observed that the risk scores possessed the greatest AUC values among the various clinically relevant prognostic factors in the TCGA cohort as well as CGGA cohorts (Fig. 4E, F). Visualization of our multivariate COX model through Nomogram to visually depict that our risk score model have a greater weight in the evaluation system (Fig. 4G, Additional file 1: Figure S3A). Moreover, to validate the accuracy of the prediction model in forecasting the prognosis of LGG patients in TCGA dataset, we conducted an analysis of time-dependent ROC curves. The AUCs at 1, 3 and 5 years were 0.86, 0.86, and 0.81, respectively (Fig. 4H, I). These findings were further supported by the results obtained from the CGGA cohort (Additional file 1: Figure S3B, S3C). Hence, exhibits the potential to accurately predict the prognosis of LGG patients across various time intervals.

### Risk model distinguishes immune landscapes and oncological characteristics of different risk groups

**Fig. 5:**
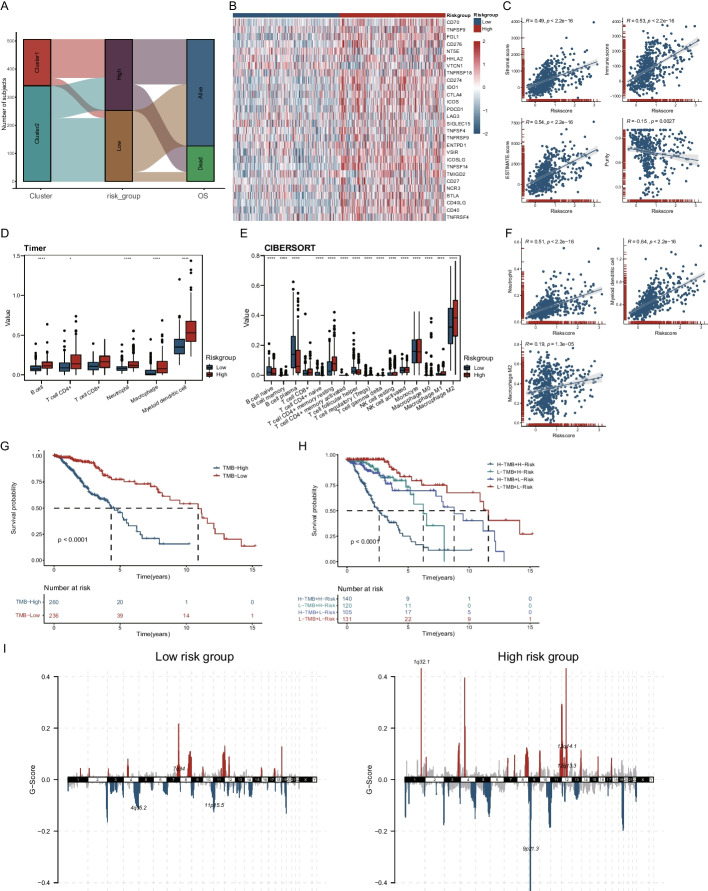
Tumor immune landscapes and tumor mutational burden between low- and high-risk subgroup in the TCGA-LGG dataset. **A** The sankey diagram of the relationship among the cluster, risk group and OS status in the TCGA-LGG patients. **B** Heatmap of the differential expression status of 29 immune checkpoint genes between low- and high-risk subgroup. **C** Dot plots show the correlation between ESTIMATE score, Stromal score, Immune score and risk score in the TCGA-LGG cohort. **D**, **E** Box plots showing differences in the infiltration of immune cells estimated using TIMER2 and CIBERSORT respectively. The Kruskal–Wallis test was used to determine the statistical significance of the difference between low- and high-risk subgroup. **F** Dot plots show the correlation between the infiltration levels of neutrophil, myeloid dendritic cell, macrophage M2 with risk score in the TCGA-LGG cohort. **G** Kaplan–Meier curve of OS time of low- and high-TMB patients in the TCGA-LGG cohort. **H** Kaplan–Meier curve of OS time of low- and high-TMB patients combined with risk score in the TCGA-LGG cohort. **I** The landscape of CNV in low- and high-risk groups

The Sankey diagram showed the correspondence between our subgroups, and the risk scores for prognostic prediction yielded similar results to the consensus clustering analysis (Fig. 5A). Then, analyzing the expression levels of immune checkpoints, CD276, CD274, CTLA4, ICOS, ICOSLG, TNFSF14, and PDCD1 were observed to be elevated in the high-risk group (Fig. 5B). In addition, the stromal score, immune score, and ESTIMATE score were all higher in the high-risk group as measured by ESTIMATE immune rating (Fig. 5C, Additional file 1: Figure S4B). With immune infiltration cell analysis, we found that B cells, CD4 + T cells, macrophages, and myeloid-derived DC cells were significantly increased among the high-risk group (Fig. 5D, E, Additional file 1: Figure S4C, D). Via a further correlation analysis, we verified that neutrophils, myeloid DC cells, and M2 macrophages were all positively correlated with risk scores (Fig. 5F, Additional file 1: Figure S4E). Combining the tumor TMB analysis, we found that patients with high TMB had shorter survival times (Fig. 5G). Additionally, risk scores remained well discriminating among patients in TMB loading groups, suggesting that patients with high-risk scores had shorter survival times regardless of whether they were classified in the high or low TMB group (Fig. 5H). Considering that tumor behavior is highly associated with mutations in genetic background, it was found by CNV analysis that the high-risk group possessed more CNVs, and a higher G-score (Fig. 5I).

In addition, the expression level of each screened lncRNA as our risk model could be an individual indicator to predict the prognosis of LGG patients (Additional file 1: Figure S5A). Mutation landscape analysis from different risk groups showed that low-risk group had a relatively higher mutation rate in IDH1(low-risk group *vs.* high-risk group: 93% *vs.* 60%), while high- risk group had a higher mutated EGFR, NF1 and PTEN gene compared with low-risk group (Additional file 1: Figure S5B). GO analysis revealed that cell functions enriched by heterogeneous genes between the high-risk group and its counterpart were as follows: regionalization, skeletal system development, pattern specification process, collagen fibril organization, anterior/posterior pattern specification (Additional file 1: Figure S5C, Additional file 2: Table S6). KEGG analysis unveiled that the signal pathways enriched by diverse genes between the high-risk group and its counterpart were: complement and coagulation cascades, ECM-receptor interaction, phagosome, focal adhesion and hematopoietic cell lineage (Additional file 1: Figure S5D).

### Predictive value of risk scores in clinical treatment subgroups

**Fig. 6:**
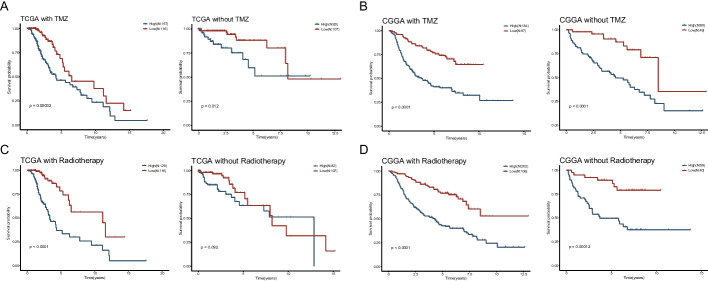
Predictive value of risk scores in clinical treatment subgroups. **A**, **B** Kaplan–Meier curves of OS for low- and high-risk groups with or without TMZ treatment in the TCGA-LGG and CGGA-LGG cohort. **C**, **D** Kaplan–Meier curves of OS for low- and high-risk groups with or without radiotherapy in the TCGA-LGG and CGGA-LGG cohort

The routine treatment of glioma includes TMZ therapy and radiotherapy. In both the TMZ-treated and non-TMZ-treated subgroups, high-risk patients had shorter survival times in the TCGA cohort (Fig. 6A). Similarly, the same findings could be obtained in the CGGA group, and the survival differences between high- and low-risk patients were more pronounced (Fig. 6B). Patients in the high-risk group had shorter survival times than those in the low-risk group when grouped according to the presence or absence of radiotherapy both in TCGA and CGGA cohorts (Fig. 6C, D).

### In vitro validation of biological functions of risk model lncRNAs

**Fig. 7:**
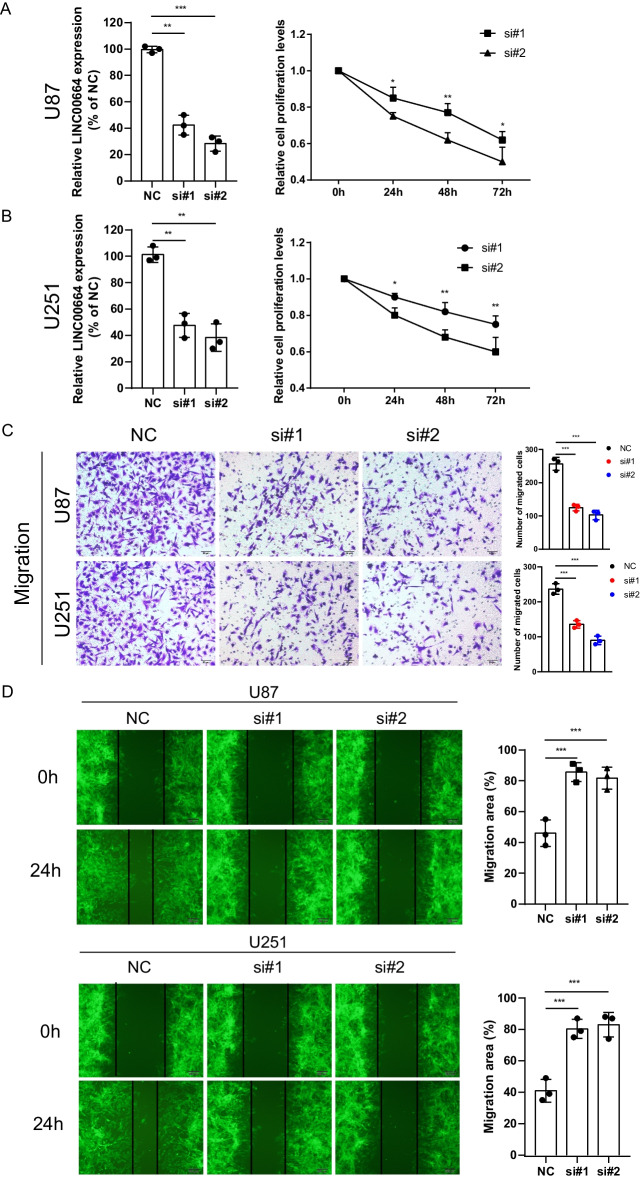
Knockdown of LINC00664 inhibited cell viability, invation and migration in glioma cells. **A**, **B** LINC00664 expression levels in U87 and U251 cells transfected with siNC or two distinct specific LINC00664 siRNAs was detected by RT-PCR. Cell viability of transfected glioma cells was detected by CCK-8 assay at 0, 24, 48, 72 h after incubation. **C** Trans-well assay were used to detect the invasion ability of U87 and U251 cells. **D** wound healing assay were used to detect the migration ability of U87 and U251 cells. The migration area means the distance between the two edges at the 24 h relative to 0 h. ***p < 0.001, **p < 0.01

LINC00664 was sorted from risk model lncRNAs to validate its biological function in two different glioma cell lines, U87 and U251. Firstly, we downregulated the expression level of LINC00664 in U87 and U251 cell lines by small interfering RNA and confirmed that it could promote the proliferation of glioma cells with the CCK8 proliferation assay (Fig. 7A, B). The relative cell proliferation levels were reduced after LINC00664 silencing in different time points (Fig. 7A, B). Compared with the negative control, at the 72 h, the lncRNA knock-down mostly reduced the cell proliferation to 62.10 ± 4.70% for si#1 and 50.21 ± 8.02% for si#2 in U87 cells, while the reduced rate was 75.01 ± 5.23% and for si#1 and 59.89 ± 8.73% for si#2 in U251 cells. Trans-well assay demonstrated that the downregulation of LINC00664 could significantly reduce the invasion ability both of U87 cells (Number of migrated cells: NC for 256.67 ± 19.09 vs. si#1 for 125.00 ± 9.85 and si#2 for 103.00 ± 14.00) and U251 cells (Number of migrated cells: NC for 236.67 ± 15.18 vs. si#1 for 136.00 ± 10.54 and si#2 for 90.00 ± 12.12) (Fig. 7C). Moreover, wound healing assay revealed that LINC00664 knock-down reduced the migration ability of U87 cells (migration area %: NC for 46.00 ± 8.54 vs. si#1 for 85.67 ± 6.11 and si#2 for 81.67 ± 7.09) and U251 cells (migration area %: NC for 41.00 ± 7.21 vs. si#1 for 80.33 ± 6.11 and si#2 for 83.00 ± 7.81) (Fig. 7D).

## Discussion

Ambulatory RNA methylation modifications, such as m6A and m5C, are involved in tumor proliferation, invasion, and immunological regulation [28]. As research progresses, there is growing evidence that lncRNAs in tumors could undergo RNA methylation and exert regulatory effects on tumors [29, 30]. A number of studies have shown that m6A and m5C methylated lncRNAs could be used as predictors of tumor survival [31, 32]. In our study, we investigated the two most common types of m6A/m5C methylated lncRNAs in LGG and constructed a predictive model for prognostic and immune characteristics. We identified that patients in the high-risk group had a shorter survival time and presented with a pro-tumor immune microenvironment including higher expression levels of immune checkpoints and suppressive immune cell infiltration. Assessment of m6A/m5C methylation-associated lncRNAs in LGG patients based on our risk model could provide innovative approaches to cluster therapy for glioma patients, while also providing a strategy for regulating the immune microenvironment in LGG.

LGG is primary brain tumors that usually occur in younger patients and have a higher long-term survival rate compared to high-grade gliomas. Therefore, a good prognostic prediction model helps us to predict and profile the survival status and tumor characteristics of patients, potentially facilitating the progress of LGG therapy and thus prolonging the survival time and quality of patients. Previously reported single-gene prediction methods no longer meet current needs, as single-gene models fail to characterize tumors adequately [33]. Zhang et al. reported a prognostic risk model for LGG based on six immune-related genes that could predict survival and immune cell infiltration [34]. Zheng et al. [35] identified three clusters of m6A RNA methylation regulators by consensus clustering to predict the prognosis of low-grade gliomas. Along with the improved understanding of functional tumor modifications and epi-transcriptomics, there is a need for a wider range of models to help us realize the relationship between LGG prognosis and functional modifications [36]. We constructed predictive models by in-depth profiling of the two most common classes of functional lncRNAs with m6A/m5C methylation functional modifications to reveal the impact of methylation lncRNA profiles in low-grade gliomas on the prognosis.lncRNAs are a class of functional RNA molecules that regulate the expression of protein-coding genes by recruiting or sequestrating gene regulatory proteins and microRNAs [37]. It was reported that LINC00689 regulated glioma cell growth, invasion, and glycolysis by competitively binding miR-338-3p to regulate the expression of the PKM2 gene [38]. Tang et al. reported that lncRNA TPTEP1 mediated MAPK signaling pathway through binding miR-106a-5p to regulate glioma stemness and radiotherapy resistance [39]. Therefore, lncRNAs are involved in the regulation of glioma from the perspective of tumor stemness maintenance, proliferation, invasion, treatment resistance, etc. In our in vitro validation, we demonstrated that one of the lncRNA LINC00664 participated in regulating the proliferation and invasion of glioma. Wang et al. reported that LINC00664 promoted the invasion, and proliferation of human oral squamous cell carcinoma via the miR-411-5p/KLF9 pathway, which was similar to our findings.

Consistent with previous research findings, the other 7 lncRNAs that we have identified in our risk model, have all been reported to play significant biological roles in gliomas, particularly HOXA-AS3, LINC00665, MIR155HG, and NEAT1. HOXA-AS3 was reported up-regulated in glioma tissue and positively related with poor prognosis in glioma patients. Knockdown of HOXA-AS3 inhibited the proliferation, invasion, and migration of GBM cells in vitro [40, 41]. LINC00665 exhibits increased expression in human glioma cell lines and tissues. Decreasing LINC00665 levels in glioma cells has been found to impede proliferation, invasion, and migration. LINC00665 acts as a ceRNA by modulating AGTR1 expression through sponging miR-34a-5p [42]. Additionally, overexpression of TAF15 stabilizes LINC00665, leading to reduced STAU1-mediated mRNA degradation of both MTF1 and YY2, thereby restricting GTSE1 transcription and ultimately disrupting glioma’s malignant progression [43]. MIR155HG has been found to be positively associated with tumor grade and represents an independent adverse prognostic factor in glioma patients. It exhibits its pro-oncogenic activity by generating miR-155-5p/-3p [44]. MIR155HG has also been implicated in promoting malignant phenotypes that enhance glioma immune evasion [45]. NEAT1 was also found up-regulation in glioma. NEAT1 promotes glioma development by upregulating SOX2 expression through the suppression of miR-132 [46]. Furthermore, NEAT1 acts as an oncogenic factor regulated by the EGFR pathway, functioning as a scaffold to recruit the chromatin modification enzyme EZH2 [47]. NEAT1 overexpression also promotes glioma progression through the stabilization of PGK1 [48].

The immune microenvironment of glioma differs significantly from other tumors outside the central nervous system. The most common immune cells in gliomas are not T cells, but macrophages, which can account for more than 20% of the tumor components [49]. Therefore, macrophages are essential contributors to the regulation of the immune microenvironment in glioma and potentially convert tumor-infiltrating T cells from active to ineffective by modulating immune checkpoints [50]. In the present study, we found a higher level of upregulated immune checkpoint expression and M2 macrophage infiltration in our high-risk group, suggesting that our model allows for the initial screening of such patients to conduct macrophage-related immunotherapy. Catalina reported that myeloid-derived suppressor cells mediate immunosuppression by transferring PD-L1 into B cells, thereby promoting the development of a tumor-suppressive microenvironment [51]. The increase in both myeloid-derived cells and B cells found in our study implied that our model would be capable of identifying glioma patients with such characteristics.

The following aspects of our study still need further improvement: 1. Lack of external real-world cohort validation; 2. Our study validated only a single lncRNA biological function in the risk model, and more comprehensive and in-depth validation is needed; 3. The predictive value of m6A/m5C methylated lncRNAs for clinical applications needs further evaluation.

## Conclusion

In this study, we constructed a prognostic prediction model of LGG by 8 m6A/m5C methylated lncRNAs and identified a critical lncRNA regulation function involved in LGG progression. High-risk patients have shorter survival times and a pro-tumor immune microenvironment.

## Supplementary Information

**Additional file 1**. Supplemental Figures.

**Additional file 2**. Supplemental Tables.

## Data Availability

This study analyzed data from The Cancer Genome Atlas (TCGA) (https://portal.gdc.cancer.gov/), Chinese Glioma Genome Atlas (CGGA) ( http://www.cgga.org.cn/) and Genotype Tissue Expression (GTEx). These data are free and publicly available.
