# Supplementary material for: The prognostic value and immune landscaps of m6A/m5C-related lncRNAs signature in the low grade glioma
Source: BMC Bioinformatics. 2023 Jul 4;24:274. doi: 10.1186/s12859-023-05386-x (PMC10320943; doi:10.1186/s12859-023-05386-x)
Supplement: Supplementary file 1 — Additional file 1. Supplemental Figures. [file 12859_2023_5386_MOESM1_ESM.pdf]

Supplemental Figure legends

Figure S1. Flow chart of this study.

Figure S2. Clustering of m6A/m5C-related lncRNAs in CGGA cohort. (A) Venn diagram show the mixed 14 lncRNAs expressed in the TCGA and CGGA datasets selected by univariate Cox analysis. (B) Principal component analysis (PCA) of cluster1 and cluster2 in the CGGA cohort. (C) Kaplan-Meier curve of overall survival time between cluster1 and cluster2 in the CGGA cohort ($p<0.0001$). (D) Heatmap of the clinicopathological features between the two distinct clusters for the CGGA cohort. (E, F) Box plots showing differences in the infiltration of immune cells estimated using TIMER2 and CIBERSORT respectively in the CGGA cohort. The Kruskal-Wallis test was used to determine the statistical significance of the difference between cluster1 and cluster2.

Figure S3. Comprehensive nomogram for m6A/m5C-related lncRNAs prognostic signature in the CGGA cohort. (A) The comprehensive nomogram predicting the clinical outcomes of LGG patients with 1-, 3- and 5-year survival based on the CGGA cohort. (B, C) ROC curve and calibration plots for predicting the 1-, 3- and 5-year OS based on the CGGA cohort.

Figure S4. Clinicopathological characteristics and tumor immune landscapes between low- and high-risk subgroup in the CGGA cohort. (A) Differences in the low- and high-risk group between various clinicalpathological features in the CGGA cohort. The Wilcoxon test was used to determine the statistical significance. (B) Dot plots show the correlation between ESTIMATE score, Stromal score, Immune score

and risk score in the CGGA cohort. (C, D) Box plots showing differences in the infiltration of immune cells estimated using TIMER2 and CIBERSORT respectively in the CGGA cohort. The Kruskal-Wallis test was used to determine the statistical significance of the difference between low- and high-risk subgroup. (E) Dot plots show the correlation between the infiltration levels of neutrophil, myeloid dendritic cell, macrophage M2 with risk score in the CGGA cohort.

Figure S5. Prognostic value of screened lncRNAs and mutation burden between low- and high-risk subgroup in the TCGA cohort. (A) Kaplan-Meier curves of OS for differential expression level of m6A/m5C-related lncRNAs prognostic signatures respectively. (B) Waterfall plots showing the distribution of the top 15 most frequent somatic mutation in the low- and high-risk subgroup in the TCGA-LGG cohort, the upper bar graph shows TMB. (C, D) GO and KEGG analysis in the low- and high-risk subgroup in the TCGA-LGG cohort.

Supplemental Table legends

Table S1. RNA methylation-related genes of m6A/m5C.

Table S2. Expression levels of 14 Screened m6A/m5C-related lncRNAs in the TCGA-LGG patients.

Table S3. Clinicopathological and clustering information in the TCGA-LGG patients.

Table S4. Construction of m6A/m5C-related lncRNAs prognostic signature in the TCGA-LGG patients.

Table S5. Clinicopathological, clustering and riskscore information in the CGGA-LGG patients.

Table S6. Differential expression genes between the high-risk group and the low-risk group in the TCGA-LGG cohort.

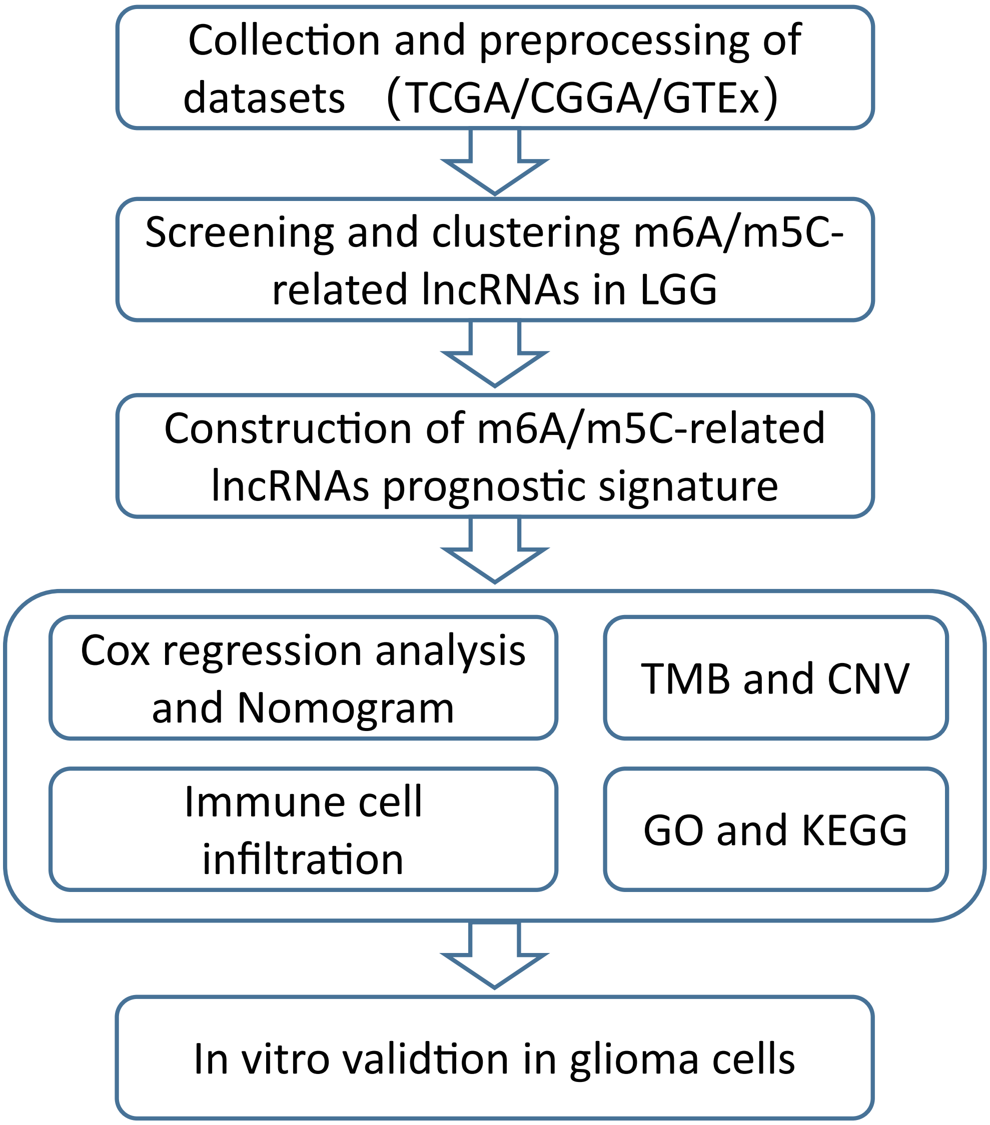
Collection and preprocessing of datasets （TCGA/CGGA/GTEx）
Screening and clustering m6A/m5C-related lncRNAs in LGG
Construction of m6A/m5C-related lncRNAs prognostic signature
Cox regression analysis and Nomogram
TMB and CNV
Immune cell infiltration
GO and KEGG
In vitro validtion in glioma cells

Fig. S1

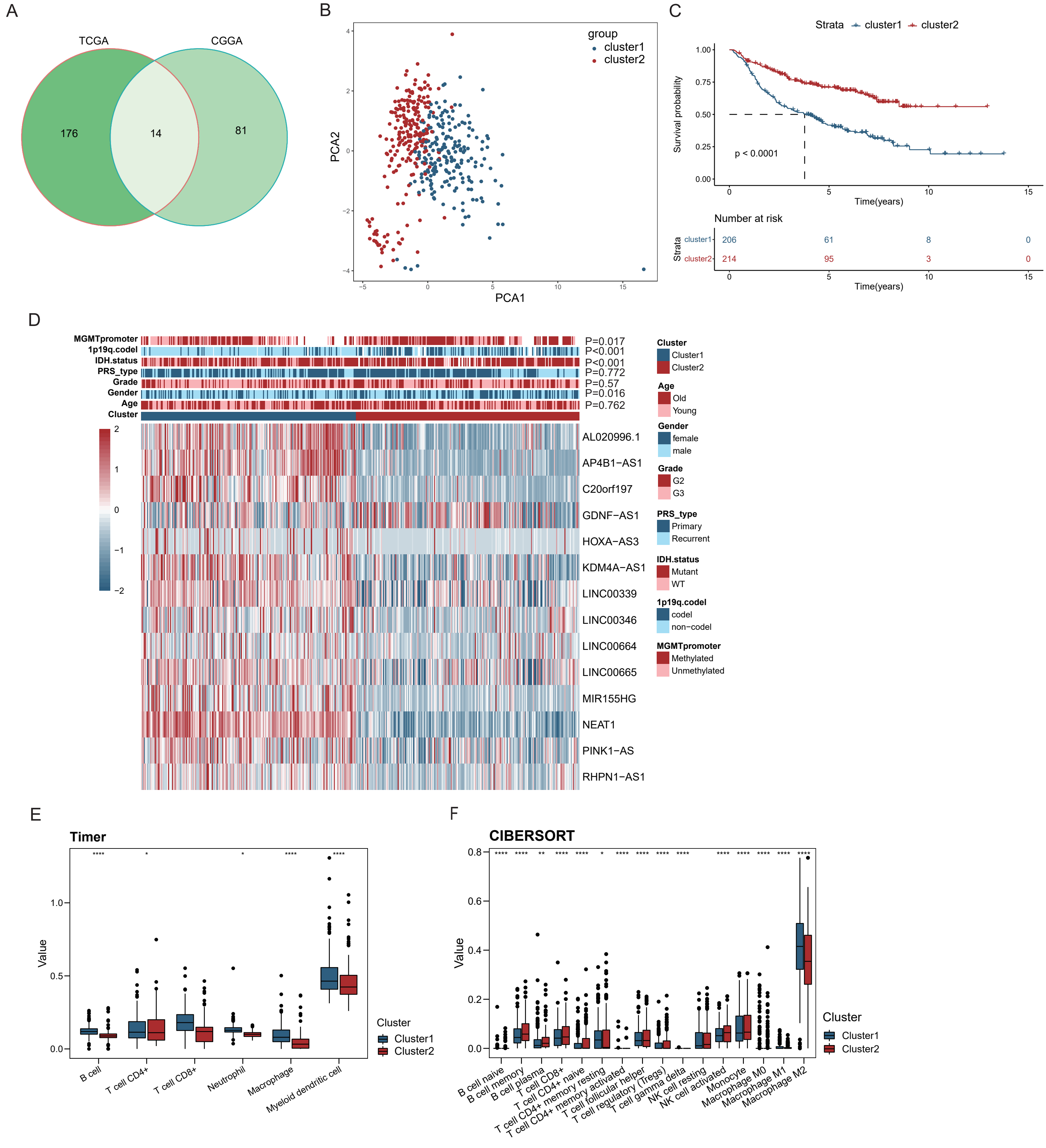
A
TCGA
CGGA
176
14
81
B
group
cluster1
cluster2
PCA1
PCA2
C
Strata
cluster1
cluster2
Survival probability
p < 0.0001
Time(years)
Number at risk
Strata
cluster1
cluster2
206
61
8
0
214
95
3
0
Time(years)
D
MGMTpromoter
1p19q.codel
IDH.status
PRS_type
Grade
Gender
Age
Cluster
P=0.017
P<0.001
P<0.001
P=0.772
P=0.57
P=0.016
P=0.762
AL020996.1
AP4B1−AS1
C20orf197
GDNF−AS1
HOXA−AS3
KDM4A−AS1
LINC00339
LINC00346
LINC00664
LINC00665
MIR155HG
NEAT1
PINK1−AS
RHPN1−AS1
Cluster
Cluster1
Cluster2
Age
Old
Young
Gender
female
male
Grade
G2
G3
PRS_type
Primary
Recurrent
IDH.status
Mutant
WT
1p19q.codel
codel
non−codel
MGMTpromoter
Methylated
Unmethylated
E
Timer
Value
B cell
T cell CD4+
T cell CD8+
Neutrophil
Macrophage
Myeloid dendritic cell
Cluster
Cluster1
Cluster2
F
CIBERSORT
Value
B cell naive
B cell memory
B cell plasma
T cell CD8+
T cell CD4+ naive
T cell CD4+ memory resting
T cell CD4+ memory activated
T cell follicular helper
T cell regulatory (Tregs)
T cell gamma delta
NK cell resting
NK cell activated
Monocyte
Macrophage M0
Macrophage M1
Macrophage M2
Cluster
Cluster1
Cluster2

Fig. S2

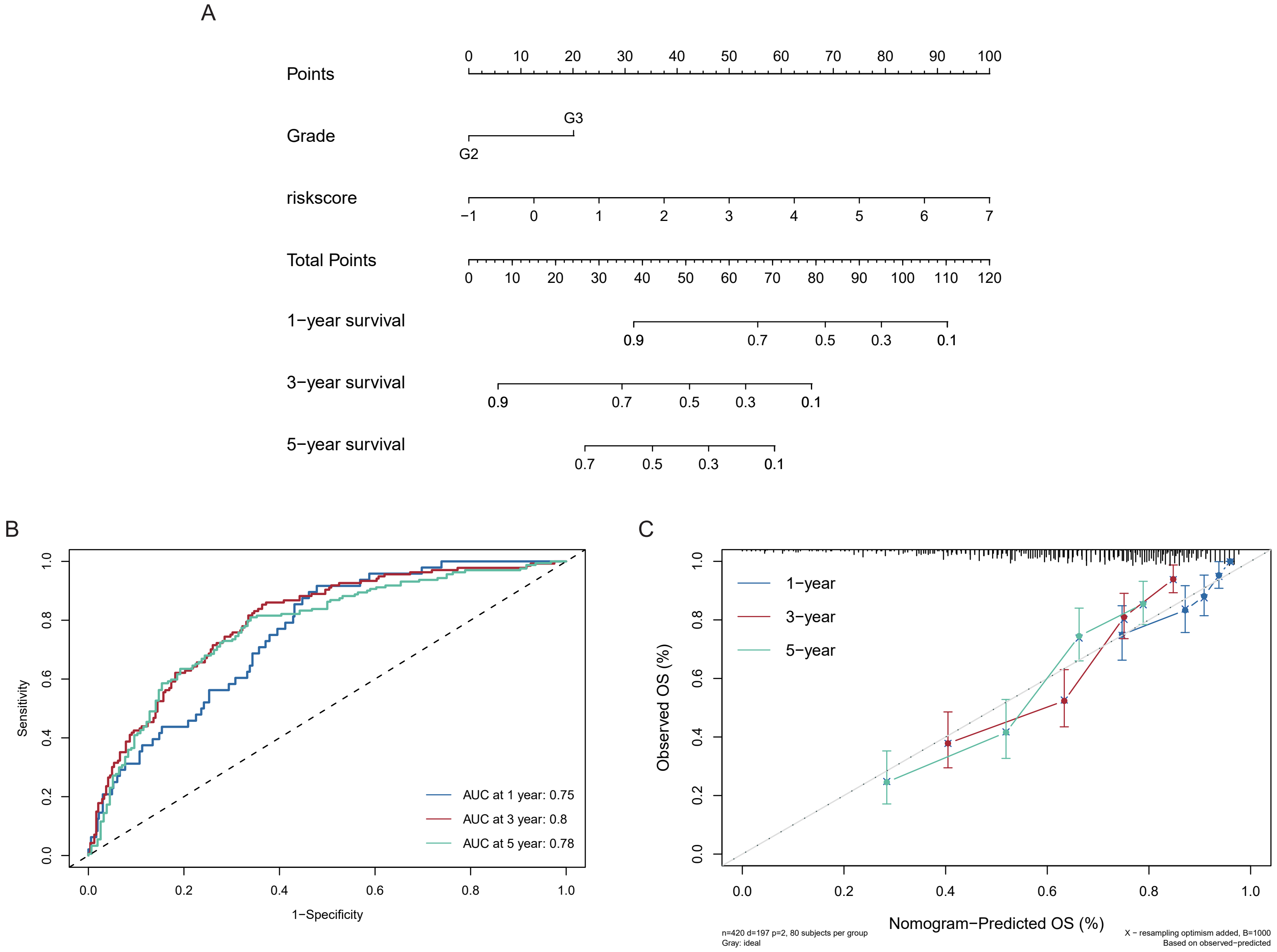
A
Points
Grade
G3
G2
riskscore
Total Points
1−year survival
3−year survival
5−year survival
B
Sensitivity
1−Specificity
AUC at 1 year: 0.75
AUC at 3 year: 0.8
AUC at 5 year: 0.78
C
Observed OS (%)
Nomogram−Predicted OS (%)
1−year
3−year
5−year
n=420 d=197 p=2, 80 subjects per group
Gray: ideal
X − resampling optimism added, B=1000
Based on observed−predicted

Fig. S3

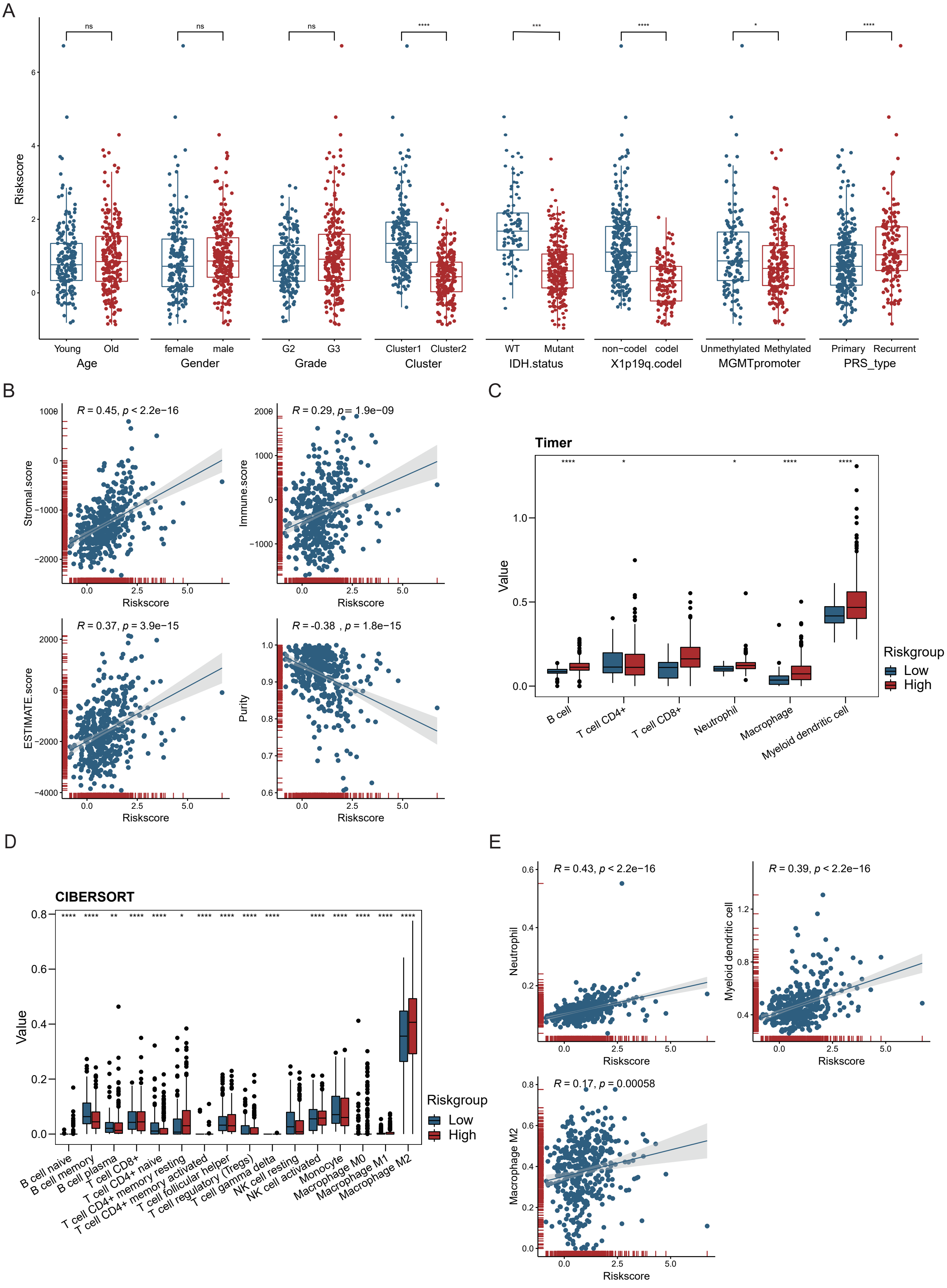
A
Riskscore
Young
Old
Age
female
male
Gender
G2
G3
Grade
Cluster1
Cluster2
Cluster
WT
Mutant
IDH.status
non−codel
codel
X1p19q.codel
Unmethylated
Methylated
MGMTpromoter
Primary
Recurrent
PRS_type
B
R = 0.45, p < 2.2e−16
Stromal.score
R = 0.29, p = 1.9e−09
Immune.score
R = 0.37, p = 3.9e−15
ESTIMATE.score
R = -0.38 , p = 1.8e−15
Purity
Riskscore
C
Timer
Value
B cell
T cell CD4+
T cell CD8+
Neutrophil
Macrophage
Myeloid dendritic cell
Riskgroup
Low
High
D
CIBERSORT
Value
B cell naive
B cell memory
B cell plasma
T cell CD8+
T cell CD4+ naive
T cell CD4+ memory resting
T cell CD4+ memory activated
T cell follicular helper
T cell regulatory (Tregs)
T cell gamma delta
NK cell resting
NK cell activated
Monocyte
Macrophage M0
Macrophage M1
Macrophage M2
Riskgroup
Low
High
E
R = 0.43, p < 2.2e−16
Neutrophil
R = 0.39, p < 2.2e−16
Myeloid dendritic cell
R = 0.17, p = 0.00058
Macrophage M2
Riskscore

Fig. S4

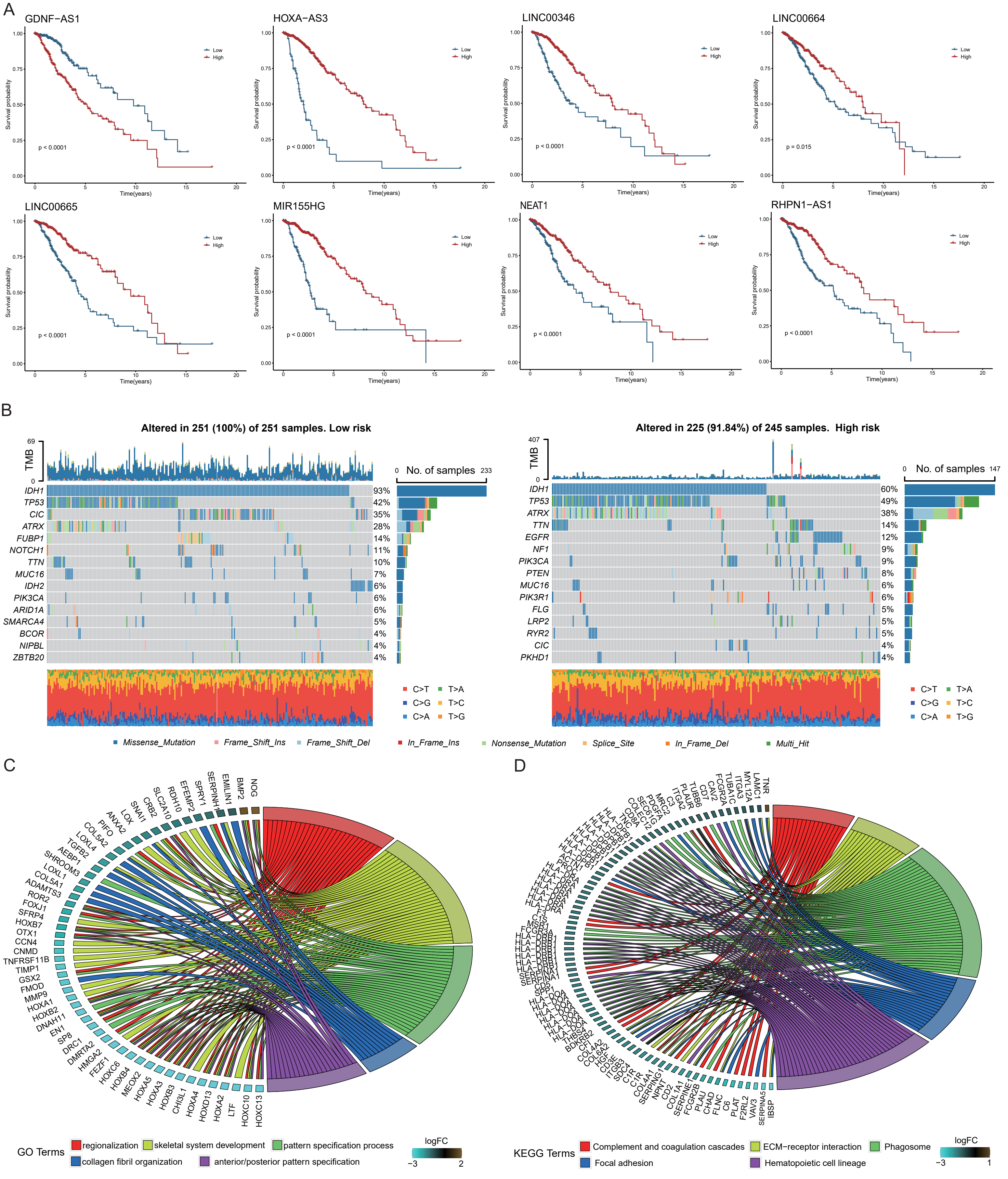

A
GDNF−AS1
HOXA−AS3
LINC00346
LINC00664
LINC00665
MIR155HG
NEAT1
RHPN1−AS1
Low
High
Survival probability
Time(years)
p < 0.0001
p = 0.015
B
Altered in 251 (100%) of 251 samples. Low risk
Altered in 225 (91.84%) of 245 samples. High risk
TMB
No. of samples
IDH1 93%
TP53 42%
CIC 35%
ATRX 28%
FUBP1 14%
NOTCH1 11%
TTN 10%
MUC16 7%
IDH2 6%
PIK3CA 6%
ARID1A 6%
SMARCA4 5%
BCOR 4%
NIPBL 4%
ZBTB20 4%
IDH1 60%
TP53 49%
ATRX 38%
TTN 14%
EGFR 12%
NF1 9%
PIK3CA 9%
PTEN 8%
MUC16 6%
PIK3R1 6%
FLG 5%
LRP2 5%
RYR2 5%
CIC 4%
PKHD1 4%
C>T
T>A
C>G
T>C
C>A
T>G
Missense_Mutation
Frame_Shift_Ins
Frame_Shift_Del
In_Frame_Ins
Nonsense_Mutation
Splice_Site
In_Frame_Del
Multi_Hit
C
NOG
BMP2
EMILIN1
SERPINH1
SPRY1
EFEMP2
RDH10
SLC2A10
CRB2
SNAI1
LOX
ANXA2
PIFO
COL5A2
LOXL4
TGFB2
AEBP1
SHROOM3
LOXL1
COL5A1
ADAMTS3
ROR2
FOXJ1
SFRP4
HOXB7
OTX1
CCN4
CNMD
TNFRSF11B
TIMP1
GSX2
FMOD
MMP9
HOXA1
HOXB2
DNAH11
EN1
SP8
DRC1
DMRTA2
HMGA2
FEZF1
HOXC6
HOXB4
MEOX2
HOXA5
HOXA3
HOXB3
CHI3L1
HOXA4
HOXD13
HOXA2
LTF
HOXC10
HOXC13
GO Terms
regionalization
skeletal system development
pattern specification process
collagen fibril organization
anterior/posterior pattern specification
logFC
−3
2
D
TNR
LAMC1
MYL12A
ITGA3
TUBA1C
FCGR2A
CAV2
CD7
TUBB6
PLAUR
ITGA2
C3
MRC2
PDGFA
SEC61G
COLEC12
CD8A
TNC
SPP1
THBS4
BDKRB2
CFI
COL4A2
COL6A2
HGF
CD36
ITGB3
SDC4
C1R
COL4A1
SERPING1
NPNT
CD2
COL1A1
SERPINE1
FCGR2B
PLAU
CHAD
FLNC
C6
PLAT
F2RL2
VAV3
SERPINA5
IBSP
KEGG Terms
Complement and coagulation cascades
ECM−receptor interaction
Phagosome
Focal adhesion
Hematopoietic cell lineage
logFC
−3
1

Fig. S5
